# Plasma exosome-derived B-cell translation gene 1: a predictive marker for the prognosis in patients with non-small cell lung cancer

**DOI:** 10.7150/jca.52320

**Published:** 2021-01-05

**Authors:** Lin Wan, Xiaochun Chen, Jun Deng, Shiliang Zhang, Fan Tu, Hao Pei, Renjing Hu, Jun Liu, Hao Yu

**Affiliations:** 1 Department of Laboratory Medicine, Wuxi Second People's Hospital, Wuxi214000, China; 2 Department of Laboratory Medicine, Taizhou Second People's Hospital, Taizhou 225300, China; 3 Department of Interventional Oncology, Wuxi Fifth People's Hospital, Wuxi214005, China; 4 Department of Laboratory Medicine, Wuxi Fifth People's Hospital, Wuxi214005, China

**Keywords:** B-cell translocation gene 1, non-small cell lung cancer, exosomal, prognosis, biomarker

## Abstract

***Objective*:** In this study, we wanted to investigate the plasma exosome-derived B-cell translocation gene 1 (BTG-1) level as a predictive marker for the prognosis in patients with Non-small cell lung cancer (NSCLC).

***Patients and Methods*:** The expression of BTG-1 protein and BTG-1 mRNA in NSCLC tissues and adjacent tissues of 98 enrolled patients were detected by immunohistochemistry (IHC), and RT-PCR. Exosome-rich fractions were isolated from the plasma of 262 NSCLC patients. ELISA was used to detect plasma exosome-derived BTG-1 levels to evaluate the predictive value for the prognosis in patients with NSCLC.

***Results*:** IHC staining showed that the positive expression rate of BTG-1 protein in NSCLC tissues was 58.16%, whereas that in adjacent tissues was 91.84%. RT-PCR showed that BTG-1 mRNA expression was significantly lower in NSCLC tissues than in adjacent tissues (52.04% vs 87.76%, *P* < 0.05). Moreover, low plasma exosome-derived BTG-1 levels were related to tumor diameter, stage, metastasis, the degree of tumor differentiation, and abnormal carcinoembryonic antigen (CEA) levels. Multivariate Cox regression analysis showed that both the disease-free survival (DFS) and overall survival (OS) were shorter in patients with low plasma exosome-derived BTG-1 level compared with patients with high plasma exosome-derived BTG-1 level.

The AUROC of plasma exosome-derived BTG-1 for 3-year DFS and 3-year OS were 0.94(95% CI; 0.91-0.98) and 0.94(95% CI: 0.90-0.98), respectively. For 3-year DFS, plasma exosome-derived BTG-1 had a sensitivity 91.0% and a specificity 82.3% for 3-year DFS, and a sensitivity 81.7% and a specificity 93.0% for 3-year OS, respectively.

***Conclusions*:** Plasma exosome-derived BTG-1 may be a potential biomarker for the prognosis in patients with NSCLC.

## Introduction

Lung cancer is a major cause of cancer-related death worldwide 1,2, causing approximately 1.6 million deaths each year 3,4. Non-small cell lung cancer (NSCLC) accounts for 85% of all primary lung cancers 5, and most patients already have advanced stage disease at the time of diagnosis, resulting in a poor prognosis 6,7. In recent years, targeted therapies and immunotherapies have shown good results in the treatment of advanced NSCLC, effectively improving patient survival 8,9. However, the presence of distant metastasis in most patients with NSCLC results in high mortality rates 10,11. Approximately 40% of new NSCLC cases exhibit metastasis at the time of diagnosis, and most patients have low 5-year survival rates. Liu et al. 12 established radiomics nomogram is a noninvasive preoperative prediction tool for malignant pulmonary nodule diagnosis. However, there was still a lack of tumor markers for early diagnosis and prognostic evaluation of NSCLC. Hence, it is important to find noninvasive, readily available, and highly specific biomarkers in the early diagnosis and prognostic evaluation of patients with NSCLC.

Members of the B-cell translocation gene (BTG) family, including BTG-1-4, Tob1, and Tob2, regulate cell growth, promote cell differentiation and maturation, and inhibit apoptosis 13-16. BTG-1 expression is regulated by p53 17 and modulates cell proliferation and differentiation. When the cell enters the proliferation cycle, BTG-1 is downregulated 18. Moreover, BTG-1 plays important roles in promoting apoptosis and suppressing the invasion and metastasis of tumor cells 19. Several studies have shown that BTG-1 expression is reduced in many tumors, including breast 20, hepatocellular 21, and ovarian cancers 22, and is closely related to the growth, metastasis, and invasion of tumors. However, the expression of BTG-1 in NSCLC has not been reported.

Exosomes are vesicular structures, which are widely distributed in blood cells, dendritic cells, tumor cells and other cells 23, 24. They can be released under physiological and pathological conditions. In recent years, a growing number of studies have reported that exocrine contains many important proteins, which can be used for early diagnosis or prognosis analysis of cancer patients 25, 26. Thus, in this study, we aimed to evaluate the levels of plasma exosome-derived BTG-1 and determine its prognostic value in patients with NSCLC.

## Materials and Methods

### Patients & clinical samples

We collected blood samples and from 262 patients with NSCLC admitted to the Wuxi Second People's Hospital, Wuxi Fifth People's Hospital and the Taizhou Second People's Hospital from December 2015 to June 2019. Of all the 262 enrolled patients, the NSCLC tissues and adjacent tissues were obtained from 98 cases. All patients underwent surgical resection. The inclusion criteria for diagnosing NSCLC were as follows: (1) NSCLC was diagnosed clinically and pathologically; (2) paraffin specimens were available for analysis; (3) patients had not undergone pre-operative radiotherapy or chemotherapy; and (4) complete clinical data were available. The exclusion criteria were as follows: (1) complicated with other malignant tumors; (2) complicated with primary brain, kidney, heart, liver, or other organ dysfunction; (3) complicated with autoimmune diseases; (4) incomplete clinical data; and (5) lost to follow up.

The baseline clinical data were collected from medical records and included demographic features (age, sex, and smoking and drinking habits), tumor characteristics (tumor size, lymph node metastasis, TNM stage, and pathological differentiation [degree of differentiation of tumor tissues, defined as similarities to normal tissues with regard to morphology and function]), and tumor markers (carcinoembryonic antigen [CEA]; abnormal CEA was defined as a CEA level > 5 ng/mL). Tumor tissues and paired adjacent tissues were isolated during surgery, fixed in 10% neutral-buffered formalin, and embedded in paraffin wax, which were used for detection of BTG-1 expression.

Patients were followed up through December 30, 2019, with a median follow-up duration of 38.5 months (range: 12.0-49.0 months). The survival data were collected from follow-up records, and disease-free survival (DFS) and overall survival (OS) were calculated. DFS was defined as the duration from resection to disease recurrence, disease progression, or death. OS was defined as the time interval from resection to death. The follow-up results for the 262 patients enrolled in this study were obtained by medical records and telephone interviews. All specimens were collected after obtaining informed consent from the patients. The study was approved by the Ethics Committees of the Taizhou Second People's Hospital (identification nos. HMU [Ethics] 2017003).

### Immunohistochemistry (IHC)

IHC was performed to detect the distribution of BTG-1 expression. An Envision and DAB chromogenic reagent kit (Antibody Diagnostic Inc., USA) was used for IHC analysis. Briefly, paraffin-embedded specimens were dewaxed in xylene and a graded series of ethanol concentrations (absolute, 95%, 85%, and 75%), and antigen retrieval was performed using citrate buffer. Next, specimens were incubated with 10% goat serum at room temperature for 30 min. The primary antibody was diluted 1:100 (sheep anti-human BTG-1 polyclonal antibodies) and was incubated with the sections for 60 min at room temperature. Sections were then incubated with secondary antibodies at room temperature for 1 h and washed with DAB solution (Jinqiao Company, China) at room temperature for 5 min. Counterstaining was performed using hematoxylin with 1% hydrochloric acid and ammonia water anti-blue for 20 s. The positive area was observed under an optical microscope, and the proportion of the positive area was calculated.

IHC results were assessed by two pathologists, and positively stained cells in NSCLC tissues and adjacent tissues were observed. Each section was randomly selected with 10 high-power fields, and 100 tumor cells were counted in each field. BTG-1 was localized in the nucleus under light microscopy. Staining scores were as follows: negative (-), there was no brown-yellow positive staining in tumors or glandular epithelial cells; weakly positive (+), the number of positive cells was less than 25%; positive (++), the number of positive cells ranged from 25% to 50%; strongly positive (+++), the number of positive cells was more than 50%. For the convenience of data statistical analysis, negative (-) and weakly positive (+) were defined as low expression, and positive (++) and strongly positive (+++) were defined as high expression.

### Detection of BTG-1 mRNA expression by RT-PCR

Total RNA was isolated from tissue using Trizol method, and was quantificated by Nandrop spectrophotometer. Total RNA (10 μg per sample) was isolated and used to generate complementary DNA. Sequences of the primers used were as follows: BTG-1: Forward: 5'-CTGCAGACCTTCAGCCAGA-3', Reverse: 5'-CGAATACAACGGTAACCCGA-3'; β-actin: 5'-TTCCAGCCTTCCTTCCTGGG-3', Reverse: 5'-TTGCGCTCAGGAGGAGGAAT-3'. It was amplified by semi quantitative polymerase chain reaction with beta-actin as reference. Thermal cycling conditions were as follows: predenaturation at 60℃ for 2 min; 40 cycles of 94℃ for 10min, 94℃ for 15s, and 62℃ for 60s. Amplication of BTG-1 by PCR was examined agarose gel electrophoresis using a Quantity-One electrophoresis apparatus. The absorbance (a) value of the belt and the reference were read, and the results were expressed by the ratio (sample value/reference value).

### Plasma exosome isolation

Remove the cells 1- 2ml human plasma (1×PBS should be diluted 5 times) with centrifuged at 500×g at 4℃ for 5 min. The supernatant, 2000×g, centrifuged at 4℃ for 10 min to remove cell debris. Large vesicles after centrifuged at 10 000×g at 4℃ for 30 min were removed. The large particles which may be mixed in the operation process was removed by filtering the supernatant with a 0.45 μm filter. The filtered supernatant was taken to the ultracentrifugation tube and 1 × PBS buffer solution was added to fill up the remaining volume to weigh and balance accurately. Put the tube on the rotor of the ultracentrifuge, centrifuge at 100000×g, 4℃ for 2 hours. The supernatant was discarded after centrifugation and at the bottom of the tube translucent sediment could be seen. The sediment was resuspended in 1×PBS buffer and centrifuged at 100 000×g at 4℃ for 80 min. 100-200 μl 1×PBS buffer was used to resuspend the exosomes which was transferred to 1.5 ml EP tube. downstream experiments can be carried out directly and stored at - 80℃ according to the requirements of follow-up experiments.

### Transmission electron microscopy (TEM)

After centrifugation,100 μ l phosphate buffer (PBS) was used to resuspend the exosome precipitate. On the copper wire mesh 20 μ l of heavy suspension was loaded. At room temperature the sample was left standing for 2 min. From the side of the filter screen the liquid was carefully absorbed with filter paper. At room temperature 20 μ l of 3% phosphotungstic acid solution was dripped into the solution and dyed. The copper mesh was then washed with double distilled water for 5 times. The samples were observed and photographed by using transmission electron microscope (Thermo-Fischer, Waltham, MA, USA) after natural drying at room temperature.

### Nanoparticle tracking analysis (NTA)

By using a pipette, the exosome suspension was evenly blown and with 1 × PBS buffer the exosome suspension was diluted 400-1000 times and at last the exosome suspension was filtered by 0.22 μ m filter to adjust to the optimal detection concentration (20-100 particals per field) of NanoSight NS300, and 1ml was injected into the instrument. The sample was irradiated with a laser (blue 488), and to record the movement of nanoparticles due to Brownian motion for 60 seconds the average frame rate of 20 frames per second was used. Each process is repeated three times. The data were output and the size distribution and particle concentration of exosomes was analyzed by NTA 3.3 software finally.

### Western blotting

For obtaining total proteins, exosomes were isolated and added to sodium dodecyl sulfate (SDS) buffer. By using SDS-PAGE gel total protein was separated and transferred onto PVDF (polyvinylidene difluoride) membranes (Millipore, USA). Membranes was blocked in 5% non-fat milk for 1 h and then incubated overnight at 4 °C with the indicated primary antibodies, including an Annexin V, TSG101, CD9 and CD63 were obtained from Santa Cruz Biotechnology, Inc., (Texas, USA). Finally, membranes were incubated by using secondary antibodies for 1 h at room temperature.

### ELISA

Residual cells were removed from the plasma sample and with 1 × PBS (1:500 dilution) cell fragments was diluted. On ice the exosomes were precipitated with 100 ml RIPA lysate for half an hour. PBS (1:3 dilution) was used to dilute the samples after shaking and mixing. The standard substance and blank control were added to the microplate and then coated with BTG-1 antibody. The diluted exosome samples were 100 μL. After incubated at 37 ℃ for 60 min, the liquid in the microplate was shook off and then pat dry the microplate, add liquid a, incubate at 37 ℃ for 60 min, wash the plate for 3 times. Add liquid B in the plate, incubate at 37 ℃ for 30 min, wash the plate for 5 times. Add 90 μL substrate and then the plate was incubated at 37 ℃ away from light for 15 min. Finally add 50 μ L of termination solution and immediately measured at 450 nm wavelength. The above methods were used to detect the level of plasma BTG-1 as a control group at the same time.

### Statistical analysis

Statistical analyses were performed with SPSS 24.0 software (IBM). Data were presented as means ± standard deviations, medians (ranges), or counts (percentages). By using McNemar's test, the expression of BTG-1 in tumor tissue and paired adjacent tissue was compared. Chi square tests or Wilcoxon's rank-sum tests was used to perform the correlation analyses. Kaplan-Meier curves showed DFS and OS. Log-rank test was used to determine the differences in DFS and OS between groups. ROC curve analysis was used to assess the prognostic value of plasma exosome-derived BTG-1 levels in osteosarcoma. Result with *P* value < 0.05was deemed to consider significant.

## Results

### Baseline characteristics

Among the 262 patients with NSCLC enrolled in this study, 195 (74.43%) were men, and 67 (25.57%) were women. Eighty-six patients (32.82%) were less than 60 years of age, and 174 patients (67.18%) were 60 years of age or older (Table 1). The numbers of patients with high, moderate, and poor tumor differentiation were 48 (18.32%), 159 (60.69%), and 55 (20.99%), respectively. Tumor size was less than 5 cm in 89 cases (40.84%) and greater than 5 cm in 155 cases (59.16%). Moreover, 99 patients (37.79%) had lymph node metastasis. In addition, 136 patients (51.91%) and 95 patients (36.26%) had a history of smoking or drinking, respectively, and 83 (31.68%), 89 (33.97%), and 90 patients (34.35%) had TNM stages I, II, and III disease, respectively. The median CEA level was 6.0 (0.8-1850.6) ng/ml.

### BTG-1 protein and BTG1 mRNA expression in NSCLC tissues and adjacent tissues

**Figure 1:**
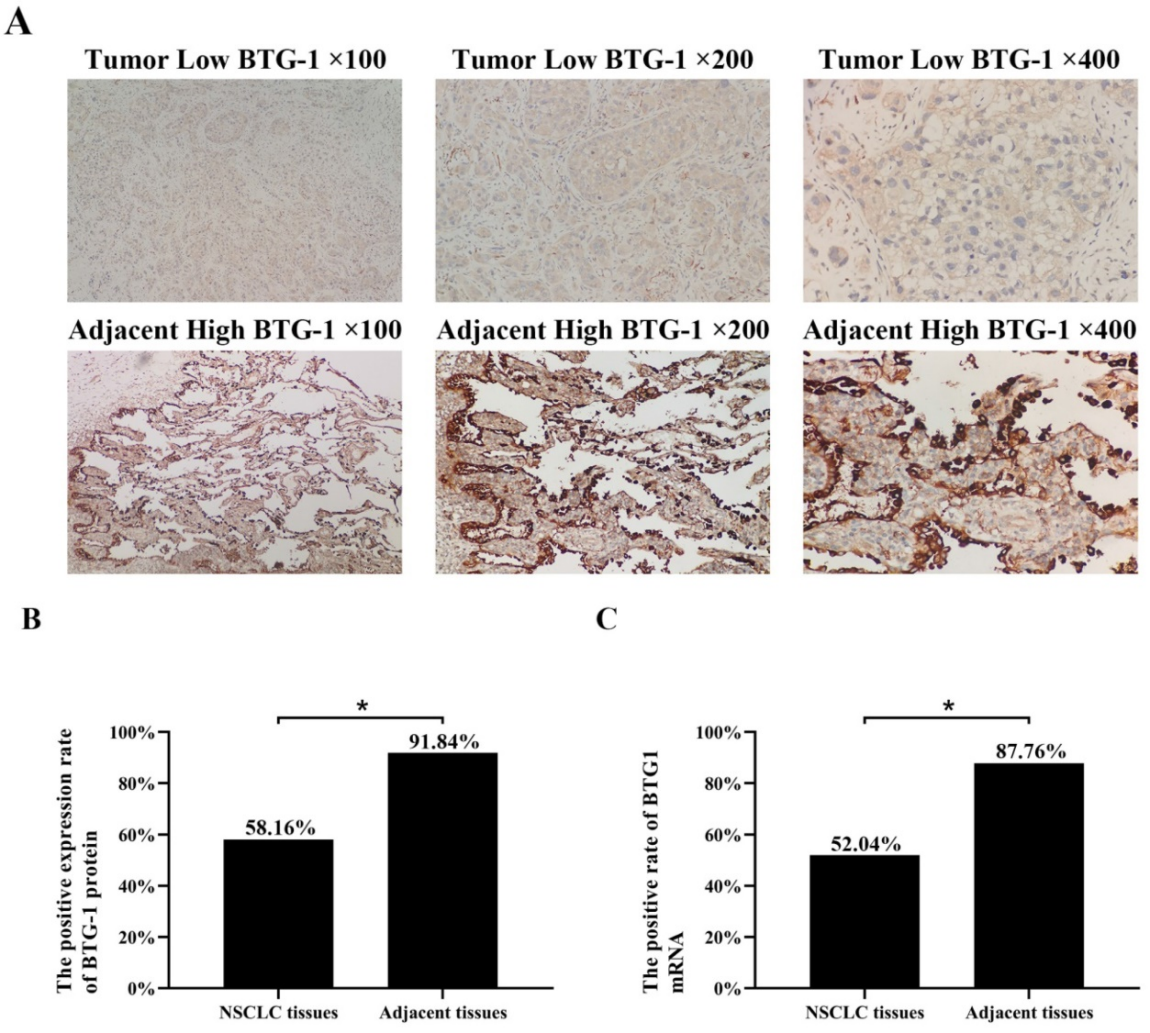
** BTG-1 protein and BTG-1 mRNA expression in NSCLC tissues and adjacent noncancerous tissues.** (A)Low BTG-1 expression in NSCLC tissues at 100×, 200×, and 400× magnification; High BTG-1 expression in adjacent tissues at 100×, 200×, and 400× magnification; (B) The positive rate of BTG-1 protein expression in NSCLC cancer tissues and adjacent tissues;(C) The positive rate of BTG-1 mRNA expression in NSCLC cancer tissues and adjacent tissues.

IHC was used to evaluate the expression of BTG-1 in NSCLC tissues and adjacent tissues. Notably, BTG-1 protein expression was significantly lower in NSCLC tissues than in adjacent tissues (Figure 1A). The positive expression rate of BTG-1 protein in NSCLC tissues was 58.16% (57/98), whereas that in adjacent tissues was 91.84% (90/98) (*P* < 0.05; Figure 1B). RT-PCR was used to detect the expression of BTG1 mRNA in the enrolled NSCLC tissues and adjacent tissues. The results showed that the positive rate of BTG1 mRNA in NSCLC was 52.04% (51/98), which was significantly lower than that in adjacent tissues 87.76% (86/98) (*P* < 0.05; Figure 1C).

### Characterization of exosomes isolated from plasma

**Figure 2:**
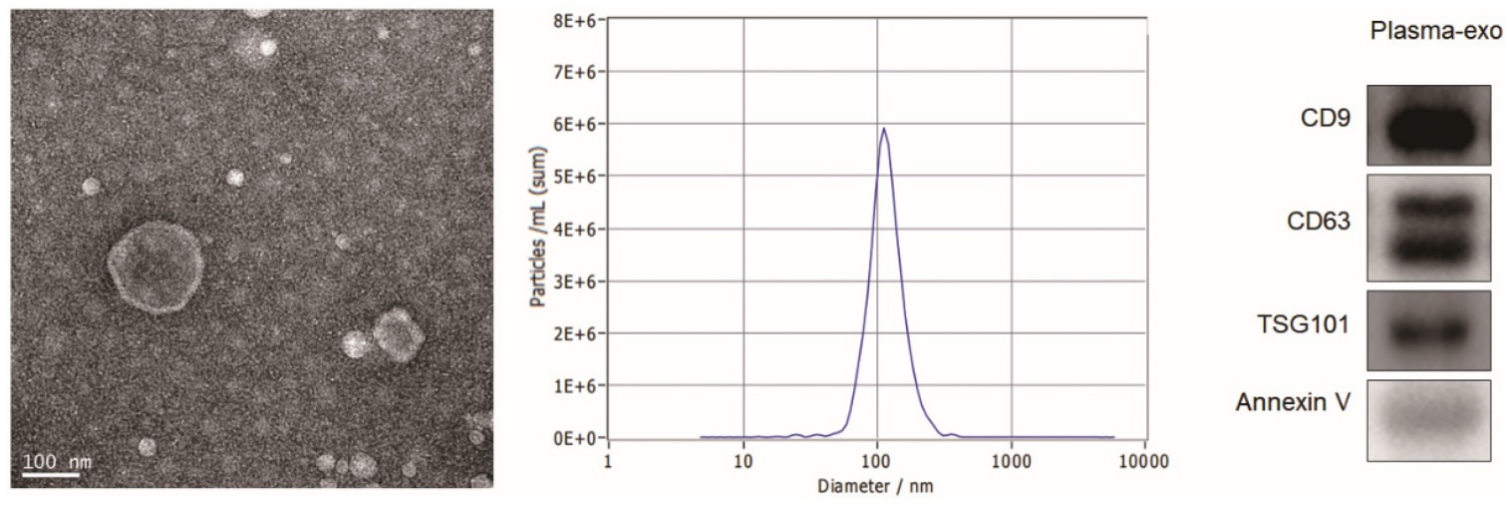
** Patient exosome characterization.** (A)TEM images showed that the exosomes were round or quasi circular vesicles with a diameter of about 40-100 nm, with complete capsule and clear background. (B)The NTA data revealed that the diameter of plasma exosome-derived BTG-1 in patients with NSCLC mainly concentrated in 60 -110 nm, and the maximum distribution peak was 102.5 nm. (C)Western blot analysis showed that the expression of exosome markers including Annexin V, Tsg101, CD9 and CD63 were found in plasma exosomes.

TEM, NTA and western blot analysis were used to confirm the exosome integrity and purification. The exosomes were obtained by gradient ultracentrifugation at low temperature and then fixed and stained. TEM images showed that the exosomes were round or quasi circular vesicles with a diameter of about 40-100 nm, with complete capsule and clear background (Figure 2A). The NTA data revealed that the diameter of plasma exosome-derived BTG-1 in patients with NSCLC mainly concentrated in 60 -110 nm, and the maximum distribution peak was 102.5 nm (Figure 2B). Western blot analysis showed that the expression of exosome markers including Annexin V, Tsg101, CD9 and CD63 were found in plasma exosomes (Figure 2C).

### Relationship between plasma exosome-derived BTG-1 levels and tumor characteristics

Next, the correlations between plasma exosome-derived BTG-1 levels and tumor characteristics in patients with NSCLC were assessed. Single factor analysis showed that low plasma exosome-derived BTG-1 levels was not related to sex or age (both *P* > 0.05), but was related to tumor diameter, stage, tumor metastasis, the degree of tumor differentiation, and abnormal CEA levels (all *P* < 0.05; Table 2).

**Figure 3:**
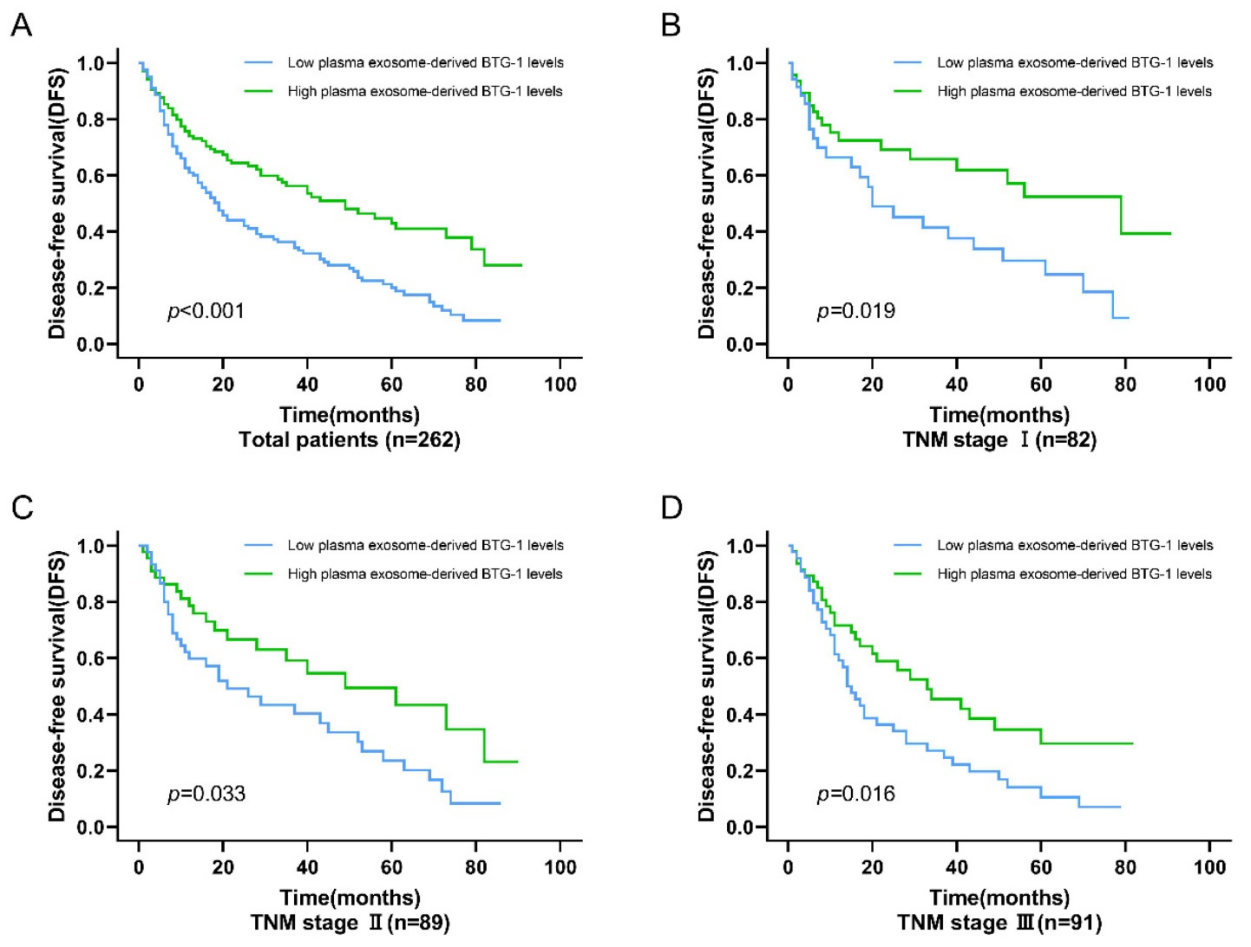
**Association of plasma exosome-derived BTG-1 level with DFS in patients with NSCLC.** (A) Among all patients with NSCLC, DFS was worse in patients who had low plasma exosome-derived BTG-1 levels compared with that in patients with high plasma exosome-derived BTG-1 levels (*P* < 0.001); (B) DFS was shorter in patients with low plasma exosome-derived BTG-1 levels than in patients with high plasma exosome-derived BTG-1 levels for those with TNM stage I (*P* = 0.019); (C) DFS was shorter in patients with low plasma exosome-derived BTG-1 levels than in patients with high plasma exosome-derived BTG-1 levels for those with TNM stage I II (*P* = 0.033); (D) DFS was shorter in patients with low plasma exosome-derived BTG-1 levels than in patients with high plasma exosome-derived BTG-1 levels for those with TNM stage III (*P* = 0.016).

Among all patients with NSCLC, DFS was worse in patients who had low plasma exosome-derived BTG-1 levels compared with that in patients with high plasma exosome-derived BTG-1 levels (*P* < 0.001; Figure 3A). Moreover, in patients with distinct TNM stages, multivariate Cox's regression analysis showed that DFS was shorter in patients with low plasma exosome-derived BTG-1 levels than in patients with high plasma exosome-derived BTG-1 levels for those with TNM stage I (*P* = 0.019), II (*P* = 0.033), and III disease (*P* = 0.016; Figure 3B-D).

**Figure 4:**
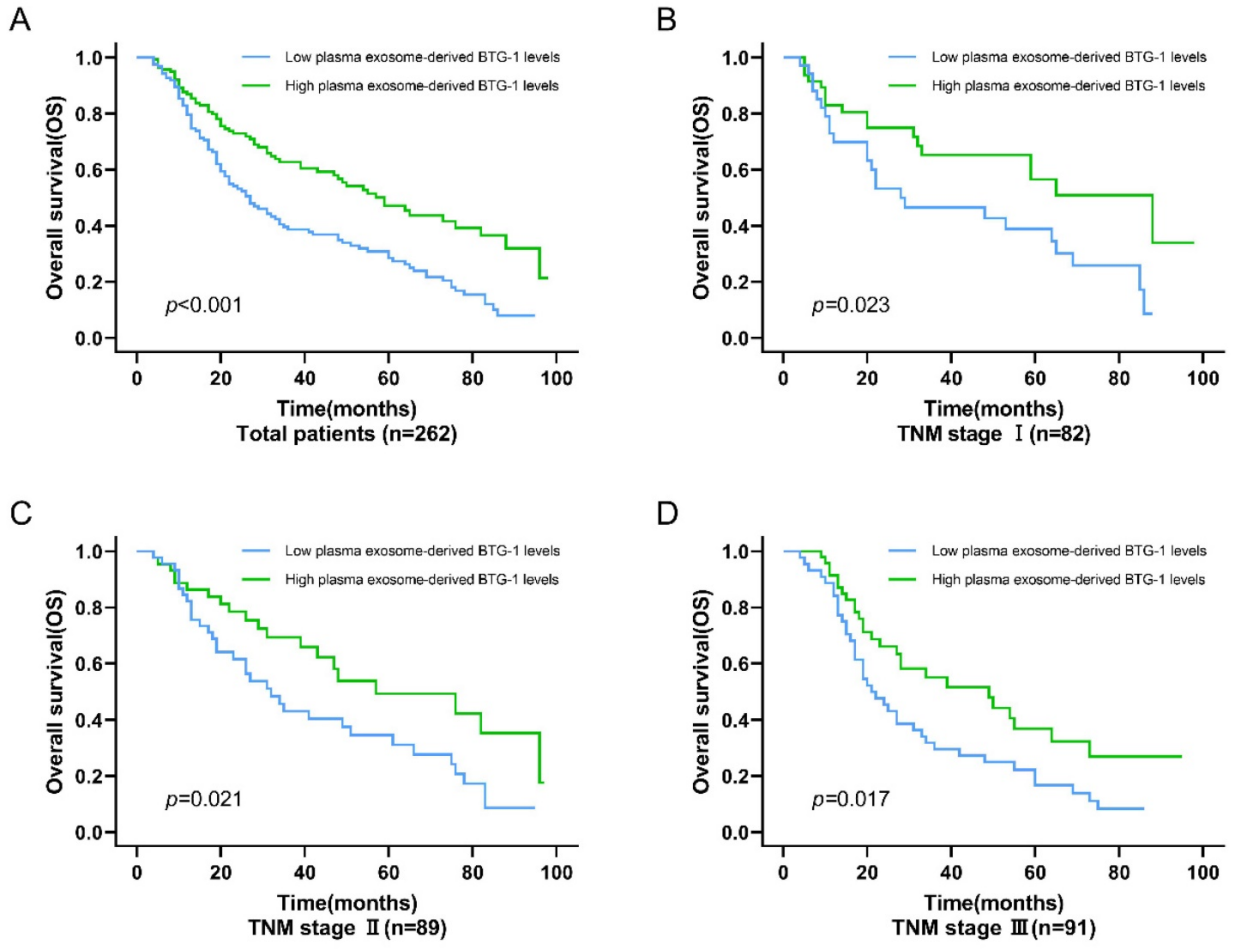
** Association of exosome-derived BTG-1 level with OS in patients with NSCLC.** (A) Among all patients, the OS of patients with low plasma exosome-derived BTG-1 levels was shorter than that in patients with high plasma exosome-derived BTG-1 levels (*P* < 0.001); (B) OS was shorter in patients with low plasma exosome-derived BTG-1 levels than in patients with high plasma exosome-derived BTG-1 levels for those with TNM stage I (*P* = 0.023); (C) OS was shorter in patients with low plasma exosome-derived BTG-1 levels than in patients with high plasma exosome-derived BTG-1 levels for those with TNM stage I II (*P* = 0.021); (D) OS was shorter in patients with low plasma exosome-derived BTG-1 levels than in patients with high plasma exosome-derived BTG-1 levels for those with TNM stage III (*P* = 0.017).

For OS, among all patients, the OS of patients with low plasma exosome-derived BTG-1 levels was shorter than that in patients with high plasma exosome-derived BTG-1 levels (*P* < 0.001; Figure 4A). Additionally, in patients with TNM stage I (*P* = 0.023; Figure 4B), II (*P* = 0.021), and III disease (*P* = 0.017; Figure 4C, D), OS was decreased in patients with low plasma exosome-derived BTG-1 levels compared with that in patients with high plasma exosome-derived BTG-1 levels.

### Prognostic value of plasma exosome-derived BTG-1 levels in patients with NSCLC

**Figure 5:**
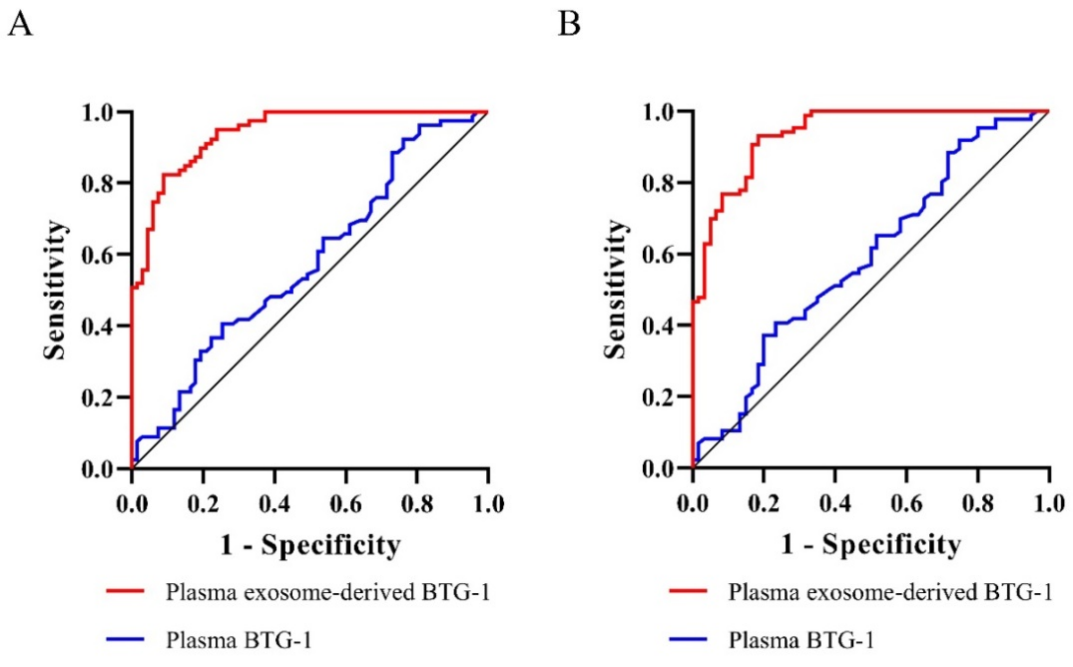
**Diagnostic power of plasma exosome-derived BTG-1 levels for the prognosis in patients with NSCLC.** (A) The AUROC of plasma exosome-derived BTG-1 for 3-year DFS was 0.94(95% CI; 0.91-0.98), while the AUROC of plasma BTG-1 was 0.58(95% CI; 0.48-0.67); (B) The AUROC of plasma exosome-derived BTG-1 for 3-year OS was 0.94(95% CI: 0.90-0.98), while the AUROC of plasma BTG-1 was 0.59(95% CI; 0.50-0.68).

We assessed the prognostic value of plasma exosome-derived BTG-1 using ROC curve analysis (Table 3). As shown (Figure 5A), the AUROC of plasma exosome-derived BTG-1, as 3-year DFS prediction biomarkers, was 0.94 (95% CI; 0.91-0.98), while the AUROC of plasma BTG-1 was 0.58 (95% CI; 0.48-0.67). With the cutoff value of 226.25, the positive predictive value, positive likelihood ratio of plasma exosome-derived BTG-1 were 81.3 (95%CI: 70.7-89.4), and 5.14 (95%CI: 3.2-8.3). The negative predictive value and negative likelihood ratio were 91.5 (95%CI: 82.5-96.8) and 0.11 (95%CI: 0.05-0.2) for prediction, with a sensitivity 91.0 (95%CI: 81.5-96.6) and a specificity 82.3 (95% CI: 72.1-90.0).

For 3-year OS, the AUROC of plasma exosome-derived BTG-1 was 0.94 (95% CI: 0.90-0.98), while the AUROC of plasma BTG-1 was 0.59 (95% CI; 0.50-0.68) (Figure 5B). With the cutoff value of 252.33, the positive predictive value, positive likelihood ratio of plasma exosome-derived BTG-1 were 89.1 (95% CI: 77.8-95.9), and 11.71 (95% CI: 5.4-25.6). The negative predictive value and negative likelihood ratio were 87.9 (95% CI: 79.4-93.8) and 0.20(95% CI: 0.1-0.3) for prediction, with a sensitivity 81.7 (95% CI: 69.6-90.5) and a specificity 93.0 (95% CI: 85.4-97.4).

## Discussion

BTG-1 is a member of the BTG/Tob antiproliferation gene family 27,28 and has been shown to block the rate of cell proliferation 29,30 by modulating the cell cycle distribution. For example, when cells are in the G_0_/G_1_ phase, BTG-1 expression is high, which can promote neovascularization and cell differentiation 31. The occurrence and development of tumors are related to unrestricted cell proliferation and reduced apoptosis rates 32. Importantly, several studies have shown that BTG-1 may induce apoptosis in tumor cells, suggesting that BTG-1 may be a tumor-suppressor gene.

In previous studies, Sheng et al. 33 showed the expression of BTG-1 protein was significantly lower in breast cancer tissue than in normal tissue. Moreover, low BTG-1 expression was significantly related to tumor invasion, lymph node metastasis, clinical stage, and histological grade in patients with breast cancer, and the OS of patients with low BTG-1 expression was shortened, suggesting a relationship between low BTG-1 expression and poor prognosis. Zhao et al. 21 showed that BTG-1 overexpression inhibited the proliferation, metastasis, and invasion of tumor cells; induced the sensitivity of tumor cells to cisplatin; and promoted G_1_ phase arrest and apoptosis. Additionally, *BTG-1* mRNA expression is negatively correlated with FIGO stage in ovarian cancer. However, the expression and biological roles of BTG-1 in NSCLC remain unclear, and also the expression and diagnosis values of BTG-1 for the prognosis in patients with NSCLC remain unclear. To date, this is the first study to examine the potential of plasma exosome-derived BTG-1 in NSCLC diagnosis.

In this study, we found that BTG-1 expression was lower in NSCLC tissues than in adjacent tissues. In order to study the role of plasma exosome-derived BTG-1 in patients with NSCLC, we extracted exosomes from the plasma of patients with NSCLC.

TEM, NTA and western blot analysis were used to confirm the exosome integrity and purification. TEM images showed that the exosomes were round or quasi circular vesicles with a diameter of about 40-100 nm, with complete capsule and clear background. The NTA data revealed that the diameter of plasma exosome-derived BTG-1 in patients with NSCLC mainly concentrated in 60 -110 nm, and the maximum distribution peak was 102.5 nm. Western blot analysis showed that the expression of exosome markers including Annexin V, Tsg101, CD9 and CD63 were found in plasma exosomes.

Next, the correlations between plasma exosome-derived BTG-1 levels and tumor characteristics in NSCLC patients were assessed. The results showed that plasma exosome-derived BTG-1 levels were related to tumor diameter, stage, tumor metastasis, the degree of tumor differentiation, and abnormal CEA levels, in accordance with previous findings for BTG-1 protein expression in other cancers. Low plasma exosome-derived BTG-1 levels were observed in the poor differentiation group and were associated with abnormal CEA levels, tumor diameter greater than or equal to 5 cm, stage III disease, and lymph node metastasis. Moreover, DFS and OS were shorter in patients with low plasma exosome-derived BTG-1 levels.

Finally, we assessed the prognostic value of plasma exosome-derived BTG-1 levels in patients with NSCLC. Plasma exosome-derived BTG-1 had higher accuracy in predicting 3-year DFS and 3-year OS. Both the sensitivity and specificity for them had good performances. For 3-year DFS, plasma exosome-derived BTG-1 had a sensitivity 91.0% and a specificity 82.3% for 3-year DFS, and a sensitivity 81.7% and a specificity 93.0% for 3-year OS, respectively. Of note, the performances of plasma exosome-derived BTG-1 were exceeded to plasma BTG-1 both in prediction for3-year DFS and 3-year OS.

This study also had several limitations which should be noted. First, although this was the largest study evaluating plasma exosome-derived BTG-1 levels in NSCLC to date, additional clinical patient validation is necessary to confirm the findings. Second, this study did not discuss plasma exosome-derived BTG-1 levels in patients with other types of lung cancer. Finally, we did not perform an assessment of the mechanisms of BTG-1 function, including up- and downstream genes, in NSCLC.

## Conclusion

In summary, our study revealed that low plasma exosome-derived BTG-1 levels were closely related to the occurrence, development, and prognosis of NSCLC. These findings may facilitate the establishment of plasma exosome-derived BTG-1 level as a novel biomarker for 3-year DFS and 3-year OS in NSCLC patients.

## Figures and Tables

**Table 1:** Baseline characteristics of enrolled NSCLC patients

Characteristic	NSCLC patients (n = 262)
Gender	
Male	195 (74.43)
Female	67 (25.57)
Age (years)	
<60	86 (32.82)
≥60	176 (67.18)
Tumor diameter (cm)	
<5	89 (40.84)
≥5	155 (59.16)
The history of smoking and drinking	
Smoke, No (%)	136 (51.91)
Drink, No (%)	95 (36.26)
Degree of tumor differentiation	
high differentiation	48 (18.32)
Moderate differentiation	159 (60.69)
poor differentiation	55(20.99)
Lymph node metastasis	
YES	99 (37.79)
NO	163 (62.21)
Tumor stage	
I	83 (31.68)
II	89 (33.97)
III	90 (34.35)
CEA (ng/ml)	6.0 (0.8-1850.6)

**Table 2:** Correlation of low plasma exosome-derived BTG-1 levels with characteristics features in NSCLC patients

Characteristic	n	Low plasma exosome-derived BTG-1 levels	x^2^	*P*
Gender, No (%)				
Male	195	117 (60.00)	0.155	0.588
Female	67	35 (52.24)		
Age (years), No (%)				
<60	86	46 (53.49)	0.172	0.673
≥60	176	106 (60.23)		
Tumor diameter (cm), No (%)				
<5	89	40 (44.94)	0.147	< 0.001
≥5	155	112 (72.26)		
Degree of tumor differentiation, No (%)				
High differentiation	48	12 (31.11)	0.233	< 0.001
Moderate differentiation	159	112 (70.44)		
Poor differentiation	55	28 (50.91)		
Lymph node metastasis, No (%)				
YES	99	65 (65.66)	0.209	0.026
NO	163	87 (53.37)		
Tumor stage, No (%)				
I	83	28 (33.73)	7.433	< 0.001
II	89	49 (55.06)		
III	90	75 (83.33)		
CEA (ng/ml), No (%)				
Normal	77	30 (38.96)	0.184	< 0.001
Abnormal	185	122 (65.95)		

**Table 3:** The prognostic value of plasma exosome-derived BTG-1 levels in NSCLC patients

Variable	NSCLC patients (n = 262)
AUROC (3-year DFS)	0.94(0.91-0.98)
Cutoff value (95%CI)	226.25
Sensitivity, %	91.0(81.5-96.6)
Specificity, %	82.3(72.1-90.0)
Positive predictive value, %	81.3(70.7-89.4)
Negative predictive value, %	91.5(82.5-96.8)
Positive likelihood ratio	5.14(3.2-8.3)
Negative likelihood ratio	0.11(0.05-0.2)
AUROC (3-year OS)	0.94(0.90-0.98)
Cutoff value (95%CI)	252.33
Sensitivity, %	81.7(69.6-90.5)
Specificity, %	93.0(85.4-97.4)
Positive predictive value, %	89.1(77.8-95.9)
Negative predictive value, %	87.9(79.4-93.8)
Positive likelihood ratio	11.71(5.4-25.6)
Negative likelihood ratio	0.20(0.1-0.3)

## Abbreviations

BTG-1: B‑cell translocation gene 1
NSCLC: non-small cell lung cancer
CEA: abnormal carcinoembryonic antigen
IHC: Immunohistochemistry
NTA: Nanoparticle tracking analysis
TEM: Transmission electron microscopy
DFS: disease-free survival
OS: overall survival
